# Convection-Enhanced Delivery: Connection to and Impact of Interstitial Fluid Flow

**DOI:** 10.3389/fonc.2019.00966

**Published:** 2019-10-02

**Authors:** Caleb A. Stine, Jennifer M. Munson

**Affiliations:** Department of Biomedical Engineering and Mechanics, Virginia Polytechnic Institute and State University, Blacksburg, VA, United States

**Keywords:** CED, glioma, brain, fluid flow, drug delivery, transport, cancer

## Abstract

Convection-enhanced delivery (CED) is a method used to increase transport of therapeutics in and around brain tumors. CED works through locally applying a pressure differential to drive fluid flow throughout the tumor, such that convective forces dominate over diffusive transport. This allows therapies to bypass the blood brain barrier that would otherwise be too large or solely rely on passive diffusion. However, this also drives fluid flow out through the tumor bulk into surrounding brain parenchyma, which results in increased interstitial fluid (IF) flow, or fluid flow within extracellular spaces in the tissue. IF flow has been associated with altered transport of molecules, extracellular matrix rearrangement, and triggering of cellular motility through a number of mechanisms. Thus, the results of a simple method to increase drug delivery may have unintended consequences on tissue morphology. Clinically, prediction of dispersal of agents via CED is important to catheter design, placement, and implementation to optimize contact of tumor cells with therapeutic agent. Prediction software can aid in this problem, yet we wonder if there is a better way to predict therapeutic distribution based simply on IF flow pathways as determined from pre-intervention imaging. Overall, CED based therapy has seen limited success and we posit that integration and appreciation of altered IF flow may enhance outcomes. Thus, in this manuscript we both review the current state of the art in CED and IF flow mechanistic understanding and relate these two elements to each other in a clinical context.

## Introduction

Convection-enhanced delivery (CED) is a technique that harnesses increased flow of fluid to increase transport of large molecules and drugs throughout a tissue. In brain cancer therapy, this technique has been implemented for decades but has not been adopted widely in the clinic. The ability of this therapy to move drugs around is useful, however there are a number of factors that can inhibit or obstruct the ability of this method to work appropriately. Fluid flow in the brain (healthy or diseased) is a constant force and it can affect not only the transport of drugs and molecules throughout the tumor and surrounding tissue, but also cause changes to tumor cells and surrounding cells that could worsen or alter disease progression. Specifically, interstitial fluid (IF) flow, or the fluid flow around cells within the porous extracellular matrix, interacts with cells to enact intracellular signaling events. CED, by its nature, increases this interstitial fluid flow (IFF) but the two are rarely discussed together. Thus, we hope to describe these flows in the context of both the natural flow in the brain and the changes in IF flow that may be attributed to the technique of CED.

### The Fluid Flow Network of the Brain: A Secondary System of Regulation

**Figure 1:**
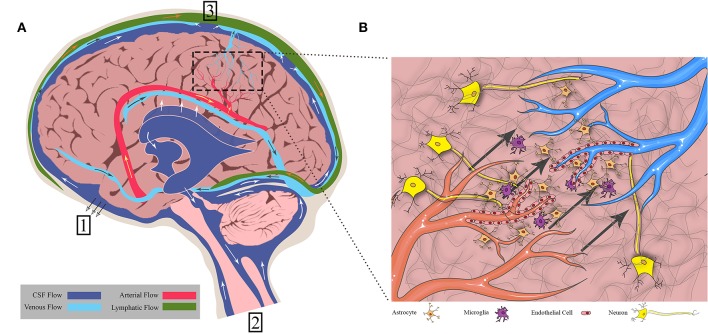
Fluid flows throughout the brain in bulk flow pathways and in interstitial space within the cellular environment. **(A)** Bulk flow pathways include CSF through the ventricles and subarachnoid space, blood through the arteries and veins, and lymph through the meningeal lymphatics. Flow direction is shown by arrows. (1) CSF to cribriform plate (2) CSF to venous sinus through arachnoid villi (3) CSF to spinal cord. **(B)** Interstitial flow moves from cerebral arterioles to venules through the endothelial cells, crossing through extracellular matrix and cells such as neurons, astrocytes, and microglia. Figure not to scale.

Within the brain fluid flow is a tightly-controlled, yet complicated, process that occurs along defined pathways. A major driver of these flows is pressure: intracranial pressure resulting from the brain incompressibility of fluid within the confined space of the cranium and hydrostatic pressure arising from circulatory dynamics. This pressure includes the tissue and fluid components of the brain and is normally around 11 mmHg (1). Pressure is regulated by the flux of bulk fluid into and out of the brain and thus is directly linked to the fluid flow pathways and rates within the tissue. Intracranial pressure changes result in shifting or compression of at least one of the four principle components of the cranial fluid vault: blood, cerebrospinal fluid (CSF), IF, and brain tissue (2). Discrete pressures can be measured in the vasculature running throughout the brain. Contraction of ventricles within the heart create this hydrostatic pressure which is the main driver of convective flow in fluid movement through the arteries and across capillary walls. Thus, this vascular pressure also drives IF flows due to the resultant pressure differential between arteries and parenchymal space. While pressure provides the force for fluid movement, anatomic structures provide the pathways (summarized in Figure 1). A fundamental understanding of these pathways and the fluids that move within them is essential to appreciate the complex effects of introducing an exogenous convective force and fluid into the brain as therapy.

### The Fluids, Structures, and Forces That Drive Flow

There are three main fluids that flow within the physiological domains of the brain: CSF, blood, and IF. Various groups have measured average flow velocities of blood, CSF, and IF (3–5). Ivanov et al. measured blood flow through cerebral capillaries in mice which was found to be 0.79 ± 0.03 mm/s. Using fluorescence intensity after bleaching on a rabbit ear, Chary and Jain measured interstitial flow to be 6 × 10^−5^ cm/s. CSF through the cerebral aqueduct was 5.27 ± 1.77 cm/s as reported by Mase et al. However, these flow rates can differ based on the localization within the tissue (i.e., major arteries vs. capillaries) and disease states.

CSF bathes the cortex and subarachnoid spaces acting as both a homeostatic regulator, distributing ions and nutrients and removing waste in the parenchyma, and as a hydraulic protector, providing buoyancy and cushioning for the brain. While there is still controversy surrounding the topic of CSF production and circulation (6, 7), general consensus is that the majority of CSF originates at the choroid plexus that line the lateral, third, and fourth ventricles (8–10). Once secreted, the CSF flows from the lateral ventricles through the interventricular foramen to the third ventricle. It then passes through the cerebral aqueduct and into the fourth ventricle before flowing into the spinal cord and subarachnoid spaces. The arachnoid villi, or arachnoid granulations, within the subarachnoid space provide a direct path for CSF to the systemic circulation through the superior sagittal sinus (11, 12). Experimental evidence suggests that another path exists through the cribriform plate. The CSF travels around olfactory nerve sheaths and is absorbed into the lymphatics within the submucosa of the olfactory epithelium (13, 14). More recently, CSF has been observed to drain into a recently (re) discovered lymphatic network within the meninges and into deep cervical lymph nodes (15, 16).

CSF flow is dynamic, driven by multiple pulsatile drivers within the central nervous system. Choroid plexus production of CSF, and subsequent velocity, has been linked to pulsatile blood flow and the cardiac cycle by Nilsson et al. (17). Phase contrast MRI indicates the pulsatile nature of CSF as it travels throughout the brain (18) indicating driving forces of heart rate (19, 20), respiration (21, 22), and ciliary beating of ependymal cells lining the ventricles and central canal of the spinal cord (23, 24). CSF flow is complicated and closely tied to other fluid movement within the brain.

Blood flow is a major driver of other fluid movement within the brain. Cerebral arteries run throughout the subarachnoid space and penetrate the cortex through the pia mater, forming what is known as the Virchow Robin space. This space is occluded from the parenchyma as the pial sheath surrounding the artery fuses with the basement membrane of the glia (6). As the arteries taper into arterioles and the complex capillary network within the parenchyma, glial cells and pericytes envelope the area around the endothelial cell layer of the blood vessels, collectively forming the blood brain barrier (BBB). Endothelial cells are especially important, forming tight junctions. The BBB limits solute transport into the brain based on size and polarity (25). The capillaries then converge to form venules and veins, leading back to the subarachnoid space, and eventually joining up with the jugular veins. To demonstrate the impact of arterial pulsation in driving transport within the brain, Rennels et al. showed that by blocking cerebral artery pulsation, horseradish peroxidase was prevented from rapid paravascular influx (20). Similarly, Hadaczek et al. infused fluorescent liposomes into rat striatum and measured distribution volumes between rats with high and low blood pressure rates (19). In rats with high blood pressure, infusate was distributed in significantly larger volumes. Thus, blood is a major driver of not only CSF flow, but also is the primary driver of endogenous IF movement.

IF, as the primary fluid within the parenchyma of the brain, is involved in cellular homeostasis and transport of nutrients. This IF is found in the spaces between the cells and extracellular matrix and is very similar in composition to CSF (26). IF originates from the blood brain barrier as the sodium-potassium pump provides a net secretion of fluid (filtered blood serum) into the parenchyma (27, 28). It may also arise as the byproduct of CSF mixing in the parenchyma as it travels via the glymphatic system.

Experimental evidence in mice suggests that CSF passes through the Virchow Robin space and enters spaces around the cerebral arteries within the cortex. In this para-arterial space, CSF passes around the astrocytic endfeet and into the interstitial space within the brain parenchyma, mixing with and becoming IF. Iliff et al. showed that IF is involved in the glymphatic system along with the CSF by injecting a tracer into the cortex and then fixing and imaging brain sections at different time points. They demonstrated that at <10 min after injection, the tracer was seen around arteries only, but after 1 h the tracer accumulated around venules as well. This indicates that IF and CSF drain via the same paravenous pathways after moving through the parenchyma. CSF and IF then collect in the corresponding paravenous spaces of the cerebral vein and, finally, either flow back to the subarachnoid space, enter the bloodstream, or drain to the cervical lymphatics (29). Interestingly, this system lines up nicely with the research done by Aspelund et al. (15) as the glymphatics would provide the link between upstream CSF and IF flow and downstream collection within the lymphatic vasculature (30). Recent criticisms debate the importance or independent existence of the glymphatic system (31, 32), but it would seem there is some means to linking the CSF and IF in the brain parenchyma. However, the degree to which they are independent vs. consistently mixed, is mostly semantic, as there are no independent barriers separating these two fluids (like with lymph and interstitial fluid for instance).

Though considered a convective force, there is supporting literature that IF flow is primarily a diffusional process (33–36) as the parenchyma has too high a hydraulic resistance for convection to occur. However, several groups have identified a convective component of IF (26, 27, 29). Abbott et al. (37) recently reviewed IF transport, which describes both contributors indicating that both convection and diffusion exist but may be dependent on anatomical location. White matter promotes convective flow as the fibers are aligned with lower amounts of dense matrix and cell bodies, whereas gray matter promotes diffusive flow (38). This has major implications for drug delivery as particles undergoing diffusion will be governed by size and particles undergoing convection will be governed by fluid flow velocity.

These pathways and fluids, in concert, offer a dynamic and complex network of flow within the brain. While we have yet to understand them altogether, significant work has been done to characterize and model the physiological state of these systems. This has led to a foundation from which abnormal flows can be studied, such as those arising in tumors, with the intention of more wholly understanding and developing therapeutic strategies against cancer.

### Disruption of Fluid Flow in Diseased States: Focus on Glioblastoma

There has been considerable work to identify the impact that diseased states have on fluid transport and how this transport can, in turn, affect disease progression. Indeed, fluid flow in the brain is dynamic along many time scales, with velocity magnitudes that fluctuate with circadian rhythm (17, 39), decrease with age (40), and vary depending on changes in blood pressure (19). Flow has also been implicated in the progression of neurological disorders such as Alzheimer's (41). But perhaps the most drastic change to flow magnitudes is from the formation of brain tumors, which will be the focus of this review with specific emphasis on glioblastoma (GBM).

GBM has an overall survival from diagnosis of <2 years, making it the deadliest primary brain tumor. This type of primary brain cancer is known for its invasive nature and most commonly arises in the cortex of the brain, specifically the frontal and temporal lobes (42). Like fluid flow, the tumor is constrained by the fundamental architecture of the brain. Microscopically, the microenvironment that these tumors grow in is a complex assortment of cells, vasculature, and extracellular matrix (ECM) that contribute to altered molecular transport and tumor progression (43). Glial and endothelial cells have been implicated in the progression of disease via invasion, maintenance of stem cell populations, and proliferation (44–50). The extracellular matrix is comprised of dense 3D networks composed of hydraulically resistant glycoproteins, proteoglycans, and hyaluronic acid (51) and contributes 20% of total brain volume (52). This fluid-rich, gel-like matrix has tortuous paths, with an estimated pore size between 20 and 60 nm that constrain and dictate the movement of molecules (36, 53). Conversely, fluid flow within the ECM can bend and stretch ECM molecules, altering the configuration of the microenvironment and triggering cellular mechanotransduction pathways (54, 55). A rich vasculature runs throughout the parenchyma yielding channels for fluid flow along glymphatic routes (56, 57).

In cancer, neo-vascularization causes a highly disorganized network of blood vessels. These vessels are also leaky due to increased permeability, are tortuous, and have blind ends (58). As blood and serum leak from the vasculature into the tumor and increase in volume, the IF pressure rises. In addition to increased fluid influx, the extracellular matrix undergoes massive reorganization by tumor cells and surrounding parenchymal cells (59, 60). This leads to decreased hydraulic conductivity and retention of fluids in the tumor bulk, further contributing to the increased IF pressure which can be as high as 45 mmHg inside some types of tumors (61). This pressure difference, specifically at the tumor border, drives flow from the tumor out into the surrounding parenchyma (62).

Dynamic contrast-enhanced imaging, which employs gadolinium contrast agents and time-lapse imaging, can be used to examine fluid movement into and within tumors. This technique is used clinically to examine blood vessel permeability and vascular transport in brain tumors. In an effort to observe the interstitial flow patterns in mouse models of glioma, (63) adapted this technique by using concentration gradients of contrast to simultaneously calculate flow velocity and diffusion, yielding a map of the flow patterns within the tumor and surrounding interstitial space. Flow directionality is heterogeneous in and around the tumor, although there are converging regions that are believed to overlap with structures (like white matter tracts) within the brain. The average interstitial flow magnitude remains relatively restricted between 0 and 6 μm/s (when corrected). D'Esposito et al. (64) created a computational model to study intratumoral IF pressure of glioma in a mouse model. They removed the tumor postmortem and cleared the tumor and cortex of the mice, imaging the vasculature afterward. This was then used in a computational model which incorporated intravascular and interstitial compartments, vascular permeability, and blood and interstitial flow to yield quantitative information about perfusion, IF pressure, and IF velocity. Findings indicate a mean IF pressure within the tumor of 16 ± 10 mmHg, an IF velocity of <0.01 μm/s in the tumor center, and an interstitial velocity of 17 ± 4 μm/s at the tumor periphery (64). Similarly, interstitial flow of tumors in general has been modeled in numerous groups (65–68) and more recently in the context of chemokine convection (69). Incorporation of these natural flows into broader models of drug delivery should allow for better prediction of drug distribution, specifically in the context of manipulating fluid flow.

## Convection-Enhanced Delivery to Drive Tissue Transport

The guiding principle behind CED is creating a positive pressure gradient to deposit treatment directly into the tumor or resection cavity and drive it through the surrounding parenchyma such that invaded cells might be accessed. This method was first described by Bobo et al. (70) in order to bypass the BBB and locally deliver chemotherapeutics or other anti-tumor agents. One such early example was the use of conjugated human transferrin to selectively target human glioma cells. Human transferrin is expressed ubiquitously in malignant tumors such as glioblastoma, but also in endothelial cells (71), creating an obstacle to intravenous delivery. CED was employed to deliver this type of drug and found to be efficacious in treating human glioma (72), eventually leading to clinical trials (73). CED uses catheters placed at specific locations to perfuse treatment directly in a, theoretically, spherical area. This method has been modeled mathematically, and at its core takes advantage of fundamental mass transport principles to increase convective over diffusive flux through the tissue.

CED is employed to solve drug delivery issues related not only to limited BBB permeability, but also to overcome high intratumoral pressures (sometimes termed the blood-tumor barrier) or limit systemic toxicity that may arise from some drugs. For example, Degen et al. conducted a study testing the dose effects of carboplatin and gemcitabine in a rat glioma model, utilizing CED or systemic delivery (74). They found that the perfusion of brain regions could be accomplished without toxicity and that the CED-treated groups had higher long-term survival. The positive pressure induced by CED drives flow through the tissue via convection-dominated transport until it reaches a certain limit governed by the infusion volume and rate. At this point, diffusion-dominated transport would govern. This means that near to the catheter, the velocity of flow is most important to the transport of the drug while farther away, the size of the drug is more important. Thus, CED is particularly beneficial to large molecule drugs, such as antibodies, nanoparticles, or conjugates (often imaging agents coupled with a drug or biomarker). Therefore, these types of therapies have been a major focus of preclinical and clinical CED application.

### CED Has Shown Limited Clinical Success

**Figure 2:**
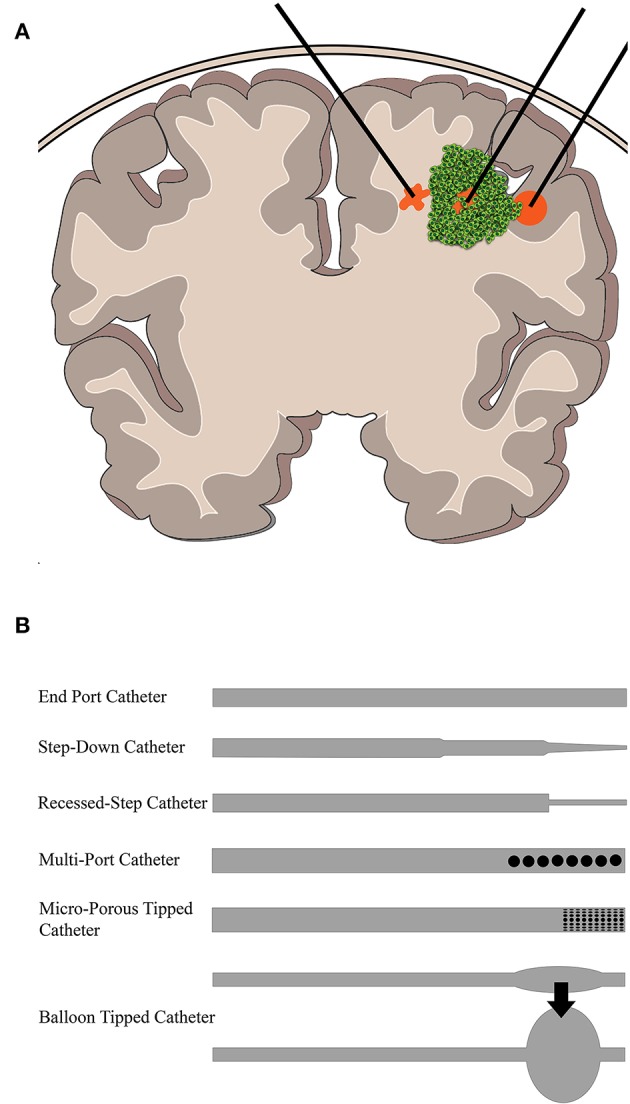
Overview of CED into brain tumors. **(A)** CED is performed through catheters placed either intratumorally or intraparenchymally. The infusate profile will change depending on region of delivery (shown in orange). **(B)** Some example catheter designs that have been used to deliver CED.

Figure 2 shows a depiction of CED at the macroscopic level as it may be clinically implemented. Infusion rates range from 0.1 to 10 μL/min, and a single catheter can usually distribute drug up to a few millimeters as confirmed by imaging (75). Clinically, the therapeutic application is defined by two terms: volume of distribution (V_d_), or amount of drug that is delivered, and volume of infusate (V_i_), the amount of infusate (drug and carrier fluid) that is delivered. The ratio of V_d_ to V_i_ is used to describe how well CED delivers a specific drug, dependent on the drug and tissue being perfused. A higher ratio is desirable as this would indicate greater distribution of drug, all else being equal. An example is the ratio of gray matter, spinal cord, and peripheral nerves which range from 4:1 to 7:1 compared to the ratio of compacted white matter which ranges from 6:1 to 10:1 (76). This means that drug distribution is greater in the white matter than the gray matter, consistent with the increased permeability of that tissue, and thus conducts fluid flow at a different rate.

Catheter design, catheter placement, tumor location, tumor size, infusion rate, infusion frequency, drug type and concentration, and brain anatomy can all contribute to differential CED responses. When working with these parameters, it is often a balance between increasing the drug distribution profile within and around the tumor and limitations on the physical implementation of CED. For instance, increasing flow rates will undoubtedly increase the distribution of drug within and around the tumor while also reducing total infusion time for the patient. However, backflow, or the tendency of the infusate to travel back up between the catheter and the tissue, is directly impacted by the infusion rate (77). Thus, oftentimes, multiple catheters have been used to better distribute and increase infusion overall at lower rates, but this can be limited by surgical access and anatomy. As such, design of catheters has been a major area of research in CED. For instance, the development of a stepped catheter design which allows CED flow rates as high as 5 μL/min in mice (78) increases flow while limiting backflow. Other new catheter designs include hollow-fiber, multi-port, ultrafine, and balloon-tipped. Lewis et al. recently reviewed the history and evolution of catheter design for CED (79). Catheter placement and infusion rate varies among the clinical trials. This is due, in part, to the more personalized approach to catheter placement necessitated by the limitations presented by an individual tumor anatomy within the brain. Further, not only are catheters placed within tumors or resection cavities, but also within the surrounding parenchyma. This variability makes it difficult to compare parameters across clinical trials using CED. To aid in some of the ambiguity of the treatment, clinical imaging is often used in conjunction with CED. Intraoperative MRI is the primary modality. By incorporating a contrast agent into the infusate or as a drug conjugate, drug distribution can be monitored and analyzed in real-time and post-treatment (80, 81).

As mentioned, infusion rates are arguably one of the most important components to CED. We will discuss later how the infusion rate is responsible for mathematically driving drug distribution. Interestingly, while this is such an important factor, it is highly variable in clinical trials to date, ranging from 0.5 μL/min to (73, 82–102) 66 μL/min (Table 1). Further, the infusion time changes substantially between independent trials. Some trials infuse for days while others only for a few hours. Some infusions are continuous while others cycle. Lastly, the total volume infused varies from 2 to 108 mL which is unsurprising given the variance in flow rates and time intervals. This lack of standardization might be one of the reasons that CED has been unable to acquire clinical success, and part of the lack of standardization is that we still do not have a holistic understanding of how CED is affected by and exerts effects on the brain and tumor tissues.

**Table 1:** Completed clinical trials of CED for human gliomas.

**Source**	**Drug (concentration)**	**Flow rate**	**Complications**	**Success/failure rate**	**Catheter placement**
Laske	Tf-CRM107 (0.1–>1 ug/ml)	0.5 uL/min increasing over 4 h to max of 4–10 ul/min for a total of 5 mL Infusion volumes increased up to 180 mL Infusions every 4–6 weeks until change seen	Reactive changes and edema (1 patient)	9/15 patients >= 50% decrease in tumor volume	1 to 3 catheters at selected sites in the tumor
Laske	Tf-CRM107 (0.67 ug/ml)	Up to 0.20 mL/h per catheter for 4–5 days until 40 mL delivered Second treatment 10 weeks after initial infusion	8/44 cerebral edema 3/44 seizure	Median survivial time 37 wks and mean survival time 45 weeks	2 catheters at selected sites in the tumor
Wersall	mAb 425	4 ml/h for 1 h	6/18 headache	Total median survival from diagnosis 39 week and from the start of mAb 18.5 week Expected median survival 24 week from start of therapy	3 to 4 catheters in the tumor-bed tissue
Rand	IL-4 pseudomonas exotoxin (0.2 μg/ml up to 6 μg/ml)	0.3–0.6 mL/h over a 4–8 day period (total infusion volume 30–185 mL)	2/9 hydrocephalus 3/9 cerebral edema	6/9 showed decreased enhancement after infusions but only one survived—the other tumors recurred	1 to 3 catheters at selected sites in the tumor based on shortest possible route. When three were used, middle inserted into center of tumor and other two placed on opposing side adjacent to largest volume of white matter
Voges	HSV-1-tk	0.025, 0.05, 0.1, 0.2, 0.4 mL/h, each at 2 h infusion time followed by 0.6 mL/h until final volume reached (30 or 60 mL)	–	Median survival time after infusion 28.1 weeks and median time to progression 8 weeks	Intracerebral
Weber	IL-4 pseudomonas exotoxin (6 μg/ml for 40 ml, 9 μg/ml for 40 ml, 15 μg/ml for 40 ml, or 9 μg/ml for 100 ml)	6.94 μL/min for 40 mL groups and 17.36 μl/min for 100 mL group. Delivered over 96 h.	26/31 seizures 10/31 (32%) cerebral edema (of those 10, 5 (50%) were serious)	Overall median survival 8.2 months with median survival of 5.8 months for GBM (highest 6-month survival for 6 μg/ml × 40 ml and 15 μg/ml × 40 ml)	1 to 3 catheters placed intratumorally
Lidar	Paclitaxel (3 patients 7.2 mg/6 mL, all others 3.6 mg/6.6 mL)	0.3 mL/h or 5 days in 24 h periods 20 cycles	2/15 edema 1/15 hydrocephalus	Median survival of 7.5 months	1 catheter placed intratumorally
Patel	Cotara (0.5–3 mCi/cm^3^)	0.18 mL/h through each catheter over 1 or 2 days (total volume 4.5–18 mL). After infusion, 0.5 mL diluent flush infused at 0.18 ml/h. 39 received first infusion, 16 received a second infusion	10/51 brain edema (20%)	–	1 to 2 catheters near or at center of enhancing tumor
Kunwar (103)	IL-13-PE38QQR (0.25–2 μg/mL for intratumoral and 0.25–1 μg/mL for intraparenchymal)	Intratumoral−0.4 or 0.54 mL/h for 48–96 h total Intraparenchymal−0.75 mL/h for 96 h to 6 days total	27 headache (53%)—catheter placmt 6 aphasia (12%)—catheter placmt 21 headache (41%)—CED of drug 10 aphasia (20%)—CED of drug	–	1–2 for intratumoral and 1–3 catheters for intraparenchymal. One cohort with intratumoral placement followed by resection and then intraparenchymal administration. One cohort with intraparenchymal placement after tumor resection
Vogelbaum (91)	IL-13-PE38QQR (0.25 or 0.5 μg/ml)	0.750 mL/h divided by # of catheters for 96 h	5 deep vein thrombosis (23%) 3 peripheral edema (14%) 3 aphasia (14%) 3 convulsion (14%)	–	2 to 4 catheters placed intraparenchymally
Sampson	TP-38 (25, 50, or 100 ng/mL)	0.4 mL/h for 50 h in each catheter (40 mL total)	Reflux and ineffective delivery in majority of patients (7/16 leak into subarachnoid space, 2/16 lead into ventricle, 4/16 pooling in necrotic area resection cavity, 3/16 successful infusion)	Overall median survival after therapy 28 weeks (20.1 for patients with residual disease and 33 for patients without residual disease)	2 catheters placed to target residual tumor or deep white matter adjacent to areas of previously resected tumor
Carpentier	CpG-ODN	0.333 mL/h for 6 h (2 mL infused total)	Seizure (5/31)	Median progression free survival 9.1 weeks and median overall survival 28 weeks	2 catheters placed intracerebrally
Kunwar (88)	IL-13-PE38QQR (0.5 μg/ml) vs. Gliadel wafers	0.75 mL/h over 96 h	10/183 brain edema 39/183 aphasia	Median survival 36.4 weeks compared to 35.4 weeks for gliadel wafers (for GBM confirmed group)	2–4 catheters placed intraparenchymally
Bruce	Topotecan (0.02, 0.04, 0.0667, 0.1, or 0.133 mg/mL)	200 μl/h in each catheter for 100 h (40 mL total)	5/18 headache 5/18 seizure	Median progression free survival 23 weeks and median overall survival 60 weeks	2 catheters placed into enhancing tumor or adjacent brain
Desjardins	Polio-rhinovirus chimera	500 μl/h over 6.5 h (3.25 mL total)	–	Median overall survival 12.5 mths compared to 11.3 mths historical and 6.6 mths NOVO-TTF-100 A treatment group	1 catheter placed intratumorally
Vogelbaum (96)	Topotecan (0.067 mg/mL)	0.396 mL/h over 96 h total (38 mL total)	–	–	2 catheters each with 4 microcatheters; 1 placed intratumorally and 1 placed intraparenchymally

Catheter placement is one of the most important steps in planning a CED intervention. Mathematical modeling and software have aided in this planning. One major factor in catheter placement is anatomical location of the tumor or location to be infused. Certain structures such as white matter tracts, ventricles, and ependymal surfaces have been known to cause failure of CED because of the impact they have on drug distribution (104, 105). The anisotropy of white matter tracts causes drug to preferentially flow through this bulk fluid pathway away from areas of therapeutic interest. Ventricles and ependymal surfaces can also act as sinks for the infusate, diminishing the Vd/Vi ratio.

Last, the drug that is delivered is extremely important to outcomes with CED and planning of infusions. Normally, when trying to deliver a drug through the vasculature and BBB, an advantage is to have it be as small as possible and potentially lipophilic so that it can pass through more easily and have a greater presence at the tumor site. CED bypasses the BBB completely, so this problem is now reversed; the drug is already where it needs to be, the issue is having it stay there. One study that examined this effect used topotecan and compared intracerebral delivery to intraperitoneal delivery using a rat glioma model (106). The authors found that the topotecan delivered systemically was able to cross the BBB, but there was a higher concentration of the topotecan in the brain and around the tumor when delivered via CED. They also observed a significant decrease in the tumor size of the CED group compared to the systemic delivery group. Because of this, drugs should have higher molecular weights and be hydrophilic if possible. Raghavan et al. (107) provide an interesting perspective into many of these clinical obstacles as well as relevant clinical scenarios in which CED could be improved upon. Some recent clinical studies with CED are highlighted in Table 1 with discussion of some of these parameters.

### CED Increases Drug Distribution in Interstitial Spaces

CED is governed by classical mass transport equations accounting for diffusive and convective flux. The changes in fluid velocity driven by CED and its impact on drug transport are best understood from this mathematical point of view. The main focus of CED is on the drug concentration profile that can be developed. This is based on the mass transport equation, which describes the change in concentration of a species over time. The general equation is dependent on diffusion and convection characteristics and is given by:

(1) $$\begin{array}{l} \frac{\partial c}{\partial t}=D\nabla^{2}c-v\cdot \nabla c+R \end{array}$$

Where the change in concentration over time ($\frac{\partial c}{\partial t}$) is solved from the diffusive component (*D*∇^2^*c*), convective component (*v*·∇*c*), and rate of any reactions taking place. In other words, transport of a species (the infusate) depends on whether it is passively diffusing, being driven by a pressure differential (bulk flow), or being replenished or depleted by chemical reactions. In the tumor microenvironment, the pressure differential between the tumor and normal tissue creates a convective force throughout the interstitial space. Depending on the species being transported by this flow, there will also be diffusion taking place (as the concentration gradient spreads out) as well as reactions between the species and surrounding cells. With regards to CED, the concentration profile is often modeled as a sphere radiating outward from the catheter tip. In this context, the mass transport equation can be written with spherical coordinates:

(2) $$\begin{array}{lll} \frac{\partial c}{\partial t} & = & D(\frac{1}{r^{2}}\frac{\partial }{\partial r}\left(r^{2}\frac{\partial c}{\partial r}\right)+\frac{1}{r^{2}sin\theta }\frac{\partial }{\partial \theta }\left(sin\theta \frac{\partial c}{\partial \theta }\right)\\ \; & \; & +\frac{1}{r^{2}\sin^{2}\theta }\frac{\partial^{2}c}{{\partial \varphi }^{2}})-v_{r}\frac{\partial c}{\partial r}-\frac{v_{\theta }}{r}\frac{\partial c}{\partial \theta }-\frac{v_{\varphi }}{rsin\theta }\frac{\partial c}{\partial \varphi }+R \end{array}$$

Where r denotes the radius of the sphere from the catheter tip, θ an angle around the tip from the z axis, and φ an angle orthogonal to θ. Together, these describe the change in concentration of infusate over time in a spherical volume.

In order to solve for the convective component of the mass transport equation, the velocity of the infusate must be known. This can be solved from the generalized Navier-Stokes equation, which defines fluid flow rate based on the properties of that fluid and the surrounding space.

(3) $$\begin{array}{l} \rho \frac{dv}{dt}+\rho v\cdot \nabla v=-\nabla P+\mu \nabla^{2}v+\rho g \end{array}$$

In this equation, the first term describes the change in velocity of the fluid over time, the second term is the convective component of the velocity, ∇*P* defines the pressure gradient, μ∇^2^*v* is the viscous or diffusive component and ρg is the effect gravity has on the velocity. Together, these terms can be used to solve for the velocity profile of a fluid. Again, because CED theoretically supplies a spherical distribution of infusate at the location of the catheter tip spherical coordinates can be used, similar to Equation 2.

In regards to the fluid flow within the tumor microenvironment, Navier-Stokes can be simplified with the assumption of incompressible, creeping flow and being a Newtonian fluid to the Stokes equation. This can then be transitioned to Darcy's law by assuming viscous forces are linear with velocity. Darcy's law describes fluid moving through a porous medium, such as flow through the interstitial space of the brain parenchyma. This is especially useful in the context of glioblastoma as the pressure differential from the tumor causes flow through the interstitial space. Darcy's law is given by:

(4) $$\begin{array}{l} q=-\frac{k}{u}\left(\frac{\Delta p}{\Delta x}\right) \end{array}$$

With this equation, the average velocity of the IF can be calculated based on the pressure differential (Δ*p*), permeability of the parenchyma (k), viscosity of the fluid (μ), and a characteristic length of tissue through which the fluid is flowing (Δx). It is important to note that this gives a superficial velocity, not a discrete profile of the flow rate. This velocity can then be paired with the mass transport equation to solve for concentration of a drug over time. It is important to note, however, that this concentration profile cannot be solved without considering the convective component which is directly tied to the interstitial flow rate that the procedure is causing as well as the impact that the surrounding tissue is causing.

One last term to consider is the Péclet number:

(5) $$\begin{array}{l} Pe=\frac{L*v}{D} \end{array}$$

Where L is the characteristic length, v is the flow velocity, and D is the mass diffusion coefficient. This term is a ratio of the convective component to the diffusive component for a given system. For a Péclet number less than one, diffusion dominates whereas a number greater than one means convection will dominate. This is important especially in the context of CED, where the main goal is to increase the convective component, by increasing the v in Equation 5, in order to obtain a larger distribution volume to the tissue. Under normal circumstances, the Péclet number in the interstitial space will be close to one, meaning that diffusion and convection components are about equal.

In the context of CED, the positive pressure induced inside the tumor from the catheter(s) would increase the pressure term in Darcy's Law, causing the velocity of the infusate through the interstitial space to increase. This velocity would also depend on the permeability of the parenchyma and tumor tissue, as the flow would have to travel through these media, and on the viscosity of the infusate. If the permeability of the tissue is higher there will be less resistance to flow, resulting in a higher flow rate as shown by Equation 4. A lower viscosity would similarly cause an increase in flow velocity, as a less viscous fluid is less resistant to deformation through shear stress. Once flow rate is determined, it can be used in the mass transport equation (v in Equation 1) to describe the convective component of drug delivery and in the Péclet number to describe how convection and diffusion are contributing. The transport of this mass is also affected by the diffusion coefficient of the drug and the reactions between the infusate and surrounding cellular environment. Together, these equations describe the drug concentration inside and around the tumor.

### Advanced Mathematical Modeling

The equations laid out in the preceding section comprise the fundamental mathematical principles that govern CED, but they have been used well before this to study fluid flow and transport in brain and other tissues (108–110). Since Bobo et al. first proposed CED, there have been numerous mathematical models trying to predict drug transport, as there are obvious clinical benefits of doing so. Early models such as that by Morrison et al. (111, 112) took into account catheter diameter, volumetric inflow rate, hydraulic flow through tissue, and deformation of the tissue and were used to model backflow. Subsequent models have built off and adapted these precursors such as Raghavan et al. (113), which reformulated and extended the model by Morrison et al. more accurately predicting backflow surrounding a cylindrical catheter based on changes in volumetric flow rate. However, more complex analytical models have been and are being created that incorporate factors such as tissue edema, fluid pathways, tissue and tumor heterogeneity, and other structural and biophysical mediators to more accurately simulate *in vivo* conditions (114–120). These models have recognized and accounted for the role that interstitial flow and structural pathways play in the CED paradigm. When examining these mathematical models, consideration of whether they are modeling CED intraparenchymally or intratumorally is important to their application. Clinically, CED can be applied into tumors alone, into tumor+parenchyma, or into resected tumor cavities or surrounding parenchyma alone. Each of these tissues presents its own set of physical parameters and challenges to planning and treatment implementation. Most of the referenced models look at perfusion into the brain tissue and not the tumor itself, which has major limitations on the results owing to the differences in mechanical and biophysical properties.

Clinically, some mathematical models have been successful. Sampson et al. (121) tested an algorithm to predict patient-specific drug distributions in a retrospective study of a CED clinical trial. The algorithm aids in placing catheters such that drugs will be delivered successfully to specific anatomical regions of the brain. The software works by first delineating fluid-filled volumes and surfaces using a T2-weighted MRI to describe the anatomy of the brain. Manual segmentation of edematous brain regions is then performed so as to not confound the algorithm. Using infusate volume and catheter dimensions, length of backflow (flow back up the outside of the catheter) is calculated and then cross-referenced to any segmented surface or cavity that is within this length. If detected, the software gives a warning of potential poor catheter placement and the catheter can be repositioned. Once verified that backflow will not occur, the fluid distribution is calculated based on the mass transport equation (Equation 1) and Darcy's law (Equation 4). The result is a patient-specific 3D profile of the drug concentration. Rosenbluth et al. (122) later refined this approach by integrating diffusion tensor imaging to include more anatomical information. Rosenbluth et al. also developed an autosegmentation tool for use with CED (123).

The use of such software offers the ability to simulate drug distribution prior to application and has helped to enhance the reproducibility of drug delivery. However, these therapeutic approaches are still not offering the expected curative outcomes for many diseases, particularly in glioblastoma. One reason for this may be the focus on distribution volume of the drug (in terms of reducing backflow and creating more targeted zones of delivery) instead of the direct impact CED has on the flow pathways within the brain. Further, there currently exists no model of CED that incorporates the naturally-occurring fluid flow within the tissue which will have a major impact on the resultant flows from an imposed pressure gradient.

## CED Directly Contributes to Increased IFF

**Figure 3:**
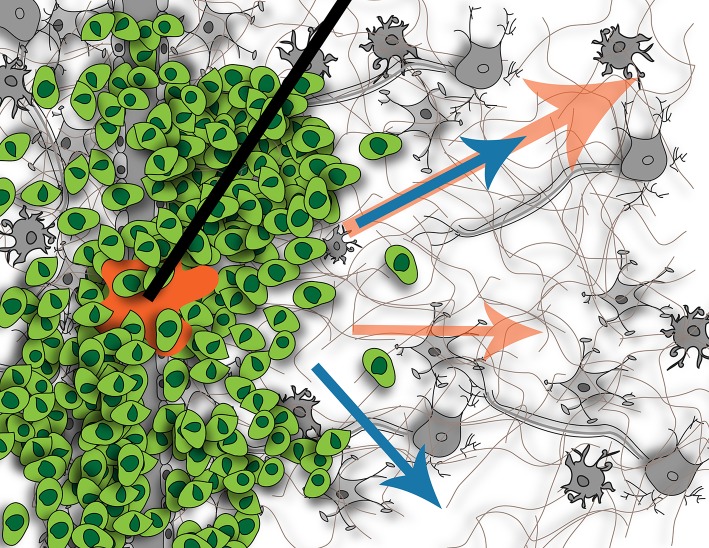
Illustration of fluid flow resulting from tumor (blue arrows) and potential effects on flow when introducing CED (orange arrows). Without CED, the tumor causes interstitial flow from its border into the surrounding parenchyma, affecting cells located there. With CED, this interstitial flow will be increased but it is not known if this will create new pathways of flow or just increase existing ones, or what the downstream impact of this increased flow will be on the resident cells. Figure not to scale.

The main focus of CED to date has been on delivering efficacious concentrations of drug in and around the tumor or resection site, but the downstream impact of this extrinsic force has not been considered. CED not only is an effort to bypass the BBB, but also to overcome the heightened intratumoral interstitial pressures. It is this same heightened pressure that drives IF flow at the tumor border into the surrounding parenchyma (61). The interstitial pressure in normal brain tissue is 0.8 mmHg, whereas it is 7 mmHg when a tumor is present (124). This increased interstitial pressure can lead to issues with CED. As mentioned, it can cause increased efflux of the CED-administered treatment back up the catheter track, reducing total delivered dose and hence, decreased clinical efficacy (80). Because CED introduces an additional hydrostatic pressure compared to a relatively normal pressure in adjacent tissue, IF flow will be induced or increased although we still don't know the downstream consequences as illustrated in Figure 3.

Like interstitial pressure, IFF rates are higher in tumor-bearing tissues as at border regions; though it can be nearly static in some regions of the tumor (63). Heightened interstitial or bulk flows can also affect CED success. In regions with low resistance to flow or along bulk fluid paths, drugs may move very quickly through the tissue reducing residence time around invaded tumor cells that are being targeted. The complex heterogeneity of tumors coupled with the anisotropy of the brain makes this balance complicated in application of flow.

CED is most often performed post-surgical resection, however, in some cases and oftentimes in canine glioma, CED is performed with the tumor in place. The inherent biophysical differences in these tissues makes exact modeling difficult, especially IF flow due to its dependence on the matrix permeability and fluid viscosity, both affected by therapeutic intervention. For instance, radiation therapy has been shown to degrade extracellular matrix in other tissues which will increase permeability of the extracellular matrix (125). As CED is most often performed on patients who have already received numerous rounds of standard of care and potentially experimental treatments, it can be difficult to generalize parameters based on healthy or even initially diagnosed patients. Strategies to use clinical imaging to identify these other properties can aid in these efforts for patient-specificity and potentially improve outcomes by identifying these parameters. Importantly, we can link the known CED derived transport to IF flow to better understand and model the effects of these changes in patients.

### IF Flow Can Drive Tumor Invasion

The effects of IF flow on cancers in general was recently reviewed by Munson and Shieh (126). In glioma, two groups have shown that this IF flow causes glioma cells to invade (126–128). The flow is thought to mediate mechanisms whereby the tumor cells actively invade the healthy tissue and may contribute to the diffusive nature of these tumors which makes them particularly difficult to cure.

Two proposed mechanisms by which flow could be mediating tumor invasion in the brain include autologous chemotaxis and cellular mechanotransduction (127). Autologous chemotaxis is the process by which a cell migrates in the direction of autologous chemokine gradients formed by IF flow carrying secreted protein upstream of the cell body (129). In glioma, the receptor, CXCR4, and its ligand, CXCL12, have been implicated in this mechanism in rat and patient glioma cells (127, 130). Mechanotransduction is the process by which cells sense and react to mechanical changes in their environment via extracellular matrix binding proteins. These cues can be induced by forces such as shear stress, compressive stress, or tensile stress (54). IF flow results in localized shear stress at the cell surface which directly signals to cytoskeletal binding proteins leading to glioma cell migration (128). One of the major receptors implicated in this mechanism is CD44 (127, 131), but other matrix-binding proteins may also be involved in mechanotransduction in the glioma microenvironment. Both CXCR4 and CD44 are highly upregulated in glioma, which further enhances the importance of studying flow in conjunction with these cancers. Paths of invasion within the brain occur in perivascular spaces around blood vessels (132), along white matter tracts (38), in perineuronal spaces, and along the meningeal layers lining the brain. Coincidentally these are regions with increased preferential bulk flow as shown by Geer and Grossman in their seminal work using convection-infused dye as a surrogate for heightened tumor pressure and tumor cells. Though these regions are subject to bulk flow as opposed to interstitial flow, much of the IF flow that is moving within the brain extracellular space eventually drains toward and along these major conduits, thus linking IF flow, bulk flow, and invasive pathways.

Recently, Cornelison et al. showed that CED therapy (at 1 μL/min) in a GL261 mouse model increased invasion of glioma cells, mediated by CXCR4. By blocking CXCR4 with AMD3100 this invasive response was effectively eliminated, suggesting that CED therapy could be more efficacious by considering the impact of fluid flow. This was the first direct proof *in vivo* that CED could lead to increased invasion. Interstitial flow in other tissues can also alter the surrounding tissues (55, 133). Interestingly, in the brains bearing GL261 tumors, not only was CXCR4 phosphorylation increased in the tumor cells with CED, but there was also observably more p-CXCR4 in the surrounding parenchymal tissue, implicating neuroglial cells have a role in possibly other flow-related signaling. These findings could have major implications on the outcome of the CED procedures, and potentially offer some partial explanation into why CED has not been shown to statistically significantly increase patient survival in clinical trials.

## Challenges and Opportunities of CED: Focus on IFF

We contend that a vital component to successful CED treatment is recognizing inherent fluid flow and pathways within the brain and their impact. Though these therapies have been implemented for decades, very few studies exist that probe the inherent contributions of the brain to CED outcomes (as opposed to CED on brain outcomes, or more often, tumor outcomes). We propose that not only are these conduits acting as passive sinks, but that the bulk fluid flow that moves along white matter tracts and within ventricles are active conduits for bulk movement of drug. Not only are these more obvious locations privy to this type of flow, but also the perineuronal or perivascular or glymphatic pathways as well. These more microscopic bulk flows offer pathways of fluid movement that can just as easily transport drug away from the tumor and quickly out of the brain. Increases in IF flow may be a good thing in this regard as keeping therapies within the interstitial spaces of the brain where they move more slowly through the complex extracellular space may offer opportunities to access more invasive cells or exert effects longer. Regardless, coupling and appreciating that there are multiple flows occurring along multiple length scales within the tissue is integral to success of a therapy that aims to alter flow. In our imaging studies, we found that though IF flow velocities were fairly consistent between animals, the intratumoral heterogeneity was high, especially in terms of direction of flow (63). Perhaps imaging flow within tissues may offer insight into CED based therapy distribution and outcomes that are not clearly apparent by simply observing the anatomy of tumor and surrounding brain.

These inherent flow pathways within the brain and natural or abnormal flows that develop due to a tumor are important when determining the appropriate design elements that are implemented. For instance, the design of catheters could account for these flow pathways by understanding the natural forces that they may be feeling beyond flow and could be designed to take advantage (coupled with placement) of inherent flows to minimize issues with backflow. Drug design and development could also take advantage of IF flow by carefully sizing particles based on the known properties of the tissue and the effect of the specific rate of IF flow within those tissues. Use of *in vitro* models of IFF in the brain (127) coupled with potential CED-based therapies could offer insight before implementation in the brain. Further, a major advance would be to continue to develop imaging modalities that can yield the parameters needed to best model fluid flow and drug distribution within individual tumors, allowing the complex computational models to better predict therapeutic delivery.

An appreciation of IF flow is also important due to its biological impact. As we mentioned, it has been shown that only a 10-fold increase in interstitial flow compared to normal physiological flow can trigger glioma cell invasion *in vitro* and *in vivo*. This response is troubling in the context of CED as the introduction of higher flows may lead to more invasion, or trigger specific invasion in already invaded tumor cells. The impact of magnitude is not yet known as IFF responses have only been studied as an on-off mechanism. A strong understanding of how tumor cells respond to heightened flows over a range is important to an understanding of the implications of CED and perhaps implementation of therapies (such as CXCR4 inhibitors or CD44 inhibitors) that can attenuate these responses. Additionally, the effect on surrounding tissues is totally unstudied, but important for a strong comprehension of how drugs may be interacting within the extracellular spaces both with other cells and with the matrix. These changes could limit drug distribution through cellular uptake and matrix binding.

In theory, CED should be a very effective treatment, if not curative. It removes many of the mysteries facing systemic delivery: known drug concentration in the tumor, defined delivery profiles, increased distribution to access invasive cells, and *in situ* and personalized treatment of patient tumors. However, in clinical trials—there has been no statistically significant difference between CED and the standard treatment modalities. This is perplexing, and we propose that there is still something we are not accounting for. Though IFF is likely not the complete picture, a better knowledge and appreciation for the inherent flows within these tissues seems one logical step to better understanding outcomes of a flow-based therapy.

## Author Contributions

JM and CS both planned, wrote, and edited the manuscript.

### Conflict of Interest

The authors declare that the research was conducted in the absence of any commercial or financial relationships that could be construed as a potential conflict of interest.
